# The association of cytomegalovirus hyperimmune globulin with adverse pregnancy outcomes

**DOI:** 10.1016/j.ajog.2025.04.014

**Published:** 2025-04-11

**Authors:** Dwight J. Rouse, Cora MacPherson, George R. Saade, Mara J. Dinsmoor, Uma M. Reddy, Robert Pass, Donna Allard, Gail Mallett, Rebecca G. Clifton, Cynthia Gyamfi-Bannerman, Michael W. Varner, William H. Goodnight, Alan T. N. Tita, Maged M. Costantine, Geeta K. Swamy, Kent D. Heyborne, Edward K. Chien, Suneet P. Chauhan, Yasser Y. El-Sayed, Brian M. Casey, Samuel Parry, Hyagriv N. Simhan, Peter G. Napolitano, George A. Macones, Brenna L. Hughes

**Affiliations:** Brown University, Providence, RI; The George Washington University Biostatistics Center, Washington, DC; The University of Texas Medical Branch, Galveston, TX; Northwestern University, Chicago, IL; *Eunice Kennedy Shriver* National Institute of Child Health and Human Development, Bethesda, MD; Department of Pediatrics, University of Alabama at Birmingham, Birmingham, AL; Brown University, Providence, RI; Northwestern University, Chicago, IL; The George Washington University Biostatistics Center, Washington, DC; Columbia University, New York, NY; The University of Utah Health Sciences Center, Salt Lake City, UT; The University of North Carolina at Chapel Hill, Chapel Hill, NC; University of Alabama at Birmingham, Birmingham, AL; The Ohio State University, Columbus, OH; Duke University, Durham, NC; University of Colorado School of Medicine, Anschutz Medical Campus, Aurora, CO; Case Western Reserve University, Cleveland, OH; Children’s Memorial Hermann Hospital, Houston, TX; Stanford University, Stanford, CA; University of Texas Southwestern Medical Center, Dallas, TX; University of Pennsylvania, Philadelphia, PA; University of Pittsburgh, Pittsburgh, PA; Madigan Army Medical Center, Joint Base Lewise–McChord, Fort Lewis, Washington; Washington University, St. Louis, MO; Brown University, Providence, RI

**Keywords:** adverse pregnancy outcomes, cytomegalovirus hyperimmune globulin

## Abstract

**OBJECTIVE:**

Because a prior randomized trial suggested that cytomegalovirus (CMV) hyperimmune globulin (HIG) might increase the frequency of adverse pregnancy outcomes,^1^ we assessed whether there was such an association in our more recent and larger trial.

**STUDY DESIGN:**

This was a secondary analysis of a multi-center randomized placebo-controlled trial of CMV HIG to prevent congenital CMV infection.^2^ Individuals were eligible for the trial if they had primary CMV infection and were carrying a singleton fetus without ultrasound abnormalities suspicious for congenital CMV infection. Participants received a monthly infusion of CMV HIG (100 mg/kg) or matching placebo until delivery. Trained research staff abstracted all outcomes according to the standard criteria. Our primary outcome for this secondary analysis was a composite of any of the following as defined in the original trial^2^: gestational hypertension (GHTN), any form of preeclampsia, small for gestational age (SGA) (birthweight<10%ile^3^), placental abruption, preterm delivery (PTD) before 37 weeks, or perinatal death. We also evaluated the association of treatment with a more severe composite outcome defined as any of the following: GHTN or preeclampsia in association with delivery before 37 weeks, birthweight<5%ile, PTD<34 weeks, or perinatal death. These composite outcomes were chosen to encompass a full range of important adverse pregnancy outcomes including those which have been previously reported in association with the use of CMV HIG,^1^ and to provide an aggregate perspective of outcomes that were reported only individually in the primary trial report.^2^ *P*<.05 was used to denote statistical significance. Statistical analysis included chi-square analysis or Fisher exact test, as appropriate, for categorical variables. All analyses were carried out using SAS software (SAS Institute Inc, Cary, NC).

**RESULTS:**

From 2012 to 2018, 399 participants were enrolled in the trial, and 390 had complete data for this analysis. Baseline characteristics were generally balanced between the treatment groups and not suggestive that those in the CMV group were at higher risk for adverse pregnancy outcome (Table). The primary adverse composite outcome was significantly more common in the CMV HIG group: 30% vs 20%; relative risk, 1.49; 95% confidence interval, 1.04 to 2.13 (Table). Though each of the individual components of the composite outcome, as well as the more severe composite outcome and its components, occurred more often in the CMV HIG group, the differences were not statistically significant (Table).

**CONCLUSION:**

We found that maternal receipt of CMV HIG was significantly associated with the frequency of a composite of GHTN, any form of preeclampsia, SGA, placental abruption, PTD, or perinatal death. Similarly, in the randomized trial of Revello et al,^1^ 7/53 (13%) of participants randomized to CMV HIG had a preterm birth, developed preeclampsia, or had a fetus diagnosed with growth restriction compared to 1/51 (2%) randomized to placebo, *P=*.06. Importantly, in neither trial did CMV HIG reduce the rate of congenital CMV infection. Why our primary outcome rate was higher is not clear. We acknowledge that both we and Revello et al have created composite outcomes that include components which are somewhat heterogenous and which may derive from differing underlying mechanisms. Nonetheless, both comprise components that are unarguably important.

This analysis was not planned and should be considered exploratory and hypothesis generating. It is of course possible that our findings arose by chance. We can only speculate that CMV HIG might engender CMV specific or nonspecific placental inflammation, apoptosis, or other processes resulting in injury or dysfunction leading to a variety of placentally mediated adverse perinatal outcomes. Antiviral antibodies when complexed with antigens combat infection through activation of the classical complement pathway, and it is possible that this activation led to destruction of the membranes of infected placental cells.^4^ However, placental pathology was not assessed in the parent study or in this secondary analysis, which is a major limitation, and data to support a relationship between the administration of immune globulin and direct placental damage are lacking. Comparisons between groups of the individual components of the primary outcome and between groups of the more severe adverse composite outcome had limited statistical power.

We present these data for 2 reasons. First, they reiterate the importance of subjecting promising treatments to randomized evaluation whenever possible, so as to get the most complete picture of the benefits and harms of the treatment, and second, for the value they may have in helping to elucidate the causes of adverse pregnancy outcomes.

## Figures and Tables

**TABLE:** Baseline characteristics

Characteristic	CMV HIG (n = 199)	Placebo (n = 191)
Maternal age, y (median [IQR])	27 [21–32]	30 [23–33]
Black (%)	18.6	15.7
Hispanic (%)	14.1	16.8
Private insurance (%)	60.3	59.2
Married or living with partner (%)	69.8	73.8
Nulliparous (%)	35.7	31.9
Prior preterm delivery (<37 wk, %)	7.0	9.4
Tobacco use (%)	11.1	12.0
Alcohol use (%)	10.6	12.0
BMI kg/m^2^ (median [interquartile range])	25.6 [22.8–30.8]	25.6 [22.1–28.8]
Chronic hypertension at baseline (%)	2.0	4.2
Diabetes	1.5	1.0
Thyroid disorder	4.5	6.3
Seizure disorder	0.5	1.6
Asthma requiring medication^a^	5.0	12.0

Outcome	No. (%)		Relative risk (95% CI)	*P* value
Primary outcome and its components				
Primary composite outcome	59 (29.6)	38 (19.9)	1.49 (1.04–2.13)	.03
GHTN^a^ or preeclampsia	24 (12.1)	14 (7.3)	1.65 (0.88–3.08)	
Birth weight<10%ile^b^	25 (12.8)	20 (10.6)	1.21 (0.69–2.09)	
Placenta abruption	3 (1.5)	1 (0.5)	2.88 (0.30–27.43)	
Preterm delivery<37 wk				
All	21 (10.6)	14 (7.3)	1.44 (0.75–2.75)	
Indicated	16 (8.0)	9 (4.7)	1.71 (0.77–3.77)	
Perinatal death	5 (2.5)	3 (1.6)	1.60 (0.39–6.60)	
Severe outcome and its components				
Severe composite outcome	29 (14.6)	18 (9.4)	1.55 (0.89–2.69)	.12
GHTN or preeclampsia and delivery before 37 wk	6 (3.0)	4 (2.1)	1.44 (0.41–5.02)	
Birth weight<5%ile^b^	20 (10.3)	10 (5.3)	1.93 (0.93–4.01)	
Preterm delivery<34 wk	7 (3.5)	5 (2.6)	1.34 (0.43–4.16)	
Perinatal death	5 (2.5)	3 (1.6)	1.60 (0.39–6.60)	

*CI*, confidence interval; *CMV*, cytomegalovirus; *GHTN*, gestational hypertension; *HIG*, hyperimmune globulin.

a *P* value=.017;

b From population standard by Alexander et al.^3^

## APPENDIX

### Trial sites and study personnel

Each clinical center consisted of 1 or more additional performance sites as listed below. Also listed are members of the *Eunice Kennedy Shriver* National Institute of Child Health and Human Development Maternal-Fetal Medicine Units Network who contributed to the research in addition to the authors.

### Clinical centers

*Brown University, Providence, RI* – B. Hughes, D. Rouse, D. Allard, E. Werner, J. Rousseau, L. Beati, J. Milano, J. Lee.

*University of Texas Medical Branch, Galveston, TX* – G. Saade, A. Salazar, L. Pacheco, J. Patel, D. Carlson, K. Smith, A. Nounes, J. DeVolder.

*Northwestern University, Chicago, IL* – G. Mallett, W. Grobman, A. Peaceman.

*NorthShore University Health System, Evanston, IL* – M. Dinsmoor, K. Paycheck.

*Columbia University, New York, NY* – C. Gyamfi-Bannerman, S. Bousleiman, R. Wapner, V. Carmona, M. Talucci.

*Christiana Care, Wilmington, DE* – M. Hoffman, A. Vanneman.

*St. Peter*’*s University Hospital, New Brunswick, NJ* – K. Palomares, C. Perez.

*Drexel University, Philadelphia, PA* – L. Plante, C. Tocci.

*New York Presbyterian Queens, Flushing, NY* – D. Skupski R. Chan-Akeley.

Lehigh Valley Hospital, Allentown, PA.

*University of Utah Health Sciences Center, Salt Lake City, UT* – M. Varner, K. Hill, A. Sowles.

*LDS Hospital, Salt Lake City, UT* – C. Meadows.

*McKay-Dee Hospital, Ogden, UT* – S. Dellerman.

*Intermountain Med Center, Salt Lake City, UT* – L. Hansen, S. Esplin.

*University of North Carolina at Chapel Hill, Chapel Hill, NC* – W. Goodnight, K. Clark, J. Thorp, S. Timlin.

*WakeMed Health & Hospitals, Raleigh, NC* – C. Beamon, H. Byers.

*Prisma Health, Greenville, SC* – K. Eichelberger, A. Moore.

*University* of *Alabama at Birmingham, Birmingham, AL* – A. Tita, S. Harris, L. Harper, J. Biggio, M. Parks, J. Sheppard.

*The Ohio State University, Columbus, OH* – M. Costantine, A. Bartholomew, M. Landon, J. Iams, C. Shellhaas, K. Markham, B. Rink, C. Buhimschi, F. Johnson, L. Webb.

*Wright State University Miami Valley Hospital, Dayton, OH* – D. McKenna, K. Fennig, K. Snow.

*Duke University, Durham, NC* – G. Swamy, T. Bishop, J. Ferrara.

*University of Colorado School of Medicine, Anschutz Medical Campus, Aurora, CO* – R. Gibbs, K. Hale, K.D. Heyborne, J. Phipers.

*MetroHealth Medical Center-Case Western Reserve University, Cleveland, OH* – E. Chien, W. Dalton.

*University Hospitals, Cleveland, OH* – D. Hackney, A. Mayle.

*UT Health- University of Texas Health Science Center at Houston, Children*’*s Memorial Hermann Hospital, Houston, TX* – S. Chauhan, F. Ortiz, B. Sibai.

*Stanford University, Stanford, CA* – Y. El-Sayed, C. Willson, N. Aziz, D. Lyell, A. Girsen, K. Sherwin.

*University of Texas Southwestern Medical Center, Dallas, TX* – B. Casey, L. Moseley, J. Price, T. Thomas, L. Fay-Randall, A. Sias, M. Garcia.

*University of Pennsylvania, Philadelphia, PA* – S. Parry, J. Craig.

*University of Pittsburgh, Pittsburgh, PA* – H. Simhan, M. Bickus, F. Facco, M. Birsic.

*Madigan Army Medical Center, Joint Base Lewis-McChord, Tacoma, WA* – P. Napolitano, L. Imbruglio, E. Hemman, J. Pates, L. Foglia.

*Data Coordinating Center: The George Washington University Biostatistics Center, Washington, DC* –E. Thom, C. MacPherson, R. Clifton, L. Fete, L. Mele, V. L. Flowers-Fanomezantsoa, T. Boekhoudt, B. Broderick, K. Barrett.

*Eunice Kennedy Shriver National Institute of Child Health and Human Development, Bethesda, MD* – U. Reddy, C. Spong, S. Pagliaro.

## References

[1] Revello MG, Lazzarotto T, Guerra B, A randomized trial of hyperimmune globulin to prevent congenital cytomegalovirus. N Engl J Med 2014;370:1316–26. 10.1056/NEJMoa1310214. 24693891

[2] Hughes BL, Clifton RG, Rouse DJ, A trial of hyperimmune globulin to prevent congenital cytomegalovirus infection. N Engl J Med 2021;385:436–44. 10.1056/NEJMoa1913569. 34320288 PMC8363945

[3] Alexander GR, Kogan M, Bader D, Carlo W, Allen M, Mor J. US birth weight/gestational age-specific neonatal mortality: 1995–1997 rates for whites, hispanics, and blacks. Pediatrics 2003;111:e61–6. 10.1542/peds.111.1.e61. 12509596 PMC1382183

[4] Goldberg BS, Ackerman ME. Antibody-mediated complement activation in pathology and protection. Immunol Cell Biol 2020;98:305–17. 32142167 10.1111/imcb.12324 PMC7293394

